# Determinants and geographic distribution of early newborn care in Ethiopia: evidence from the 2019 Ethiopian Mini Demographic Health Survey

**DOI:** 10.1038/s41598-023-49812-9

**Published:** 2023-12-20

**Authors:** Daniel G. Belay, Melaku Birhanu Alemu, Gavin Pereira, Zohra S. Lassi, Gizachew A. Tessema

**Affiliations:** 1 Department of Epidemiology and Biostatistics, Institute of Public Health, College of Medicine and Health Sciences, University of Gondar, Gondar, Ethiopia; 2 Department of Health Systems and Policy, Institute of Public Health, College of Medicine and Health Sciences, University of Gondar, Gondar, Ethiopia; 3 Curtin School of Population Health, Curtin University, Perth, WA Australia; 4 enAble Institute, Curtin University, Perth, Kent Street, Bentley, Perth, WA Australia; 5 WHO Collaborating Centre for Environmental Health Impact Assessment, Faculty of Health Science, Curtin University, Bentley, Perth, WA Australia; 6 School of Public Health, University of Adelaide, Adelaide, SA Australia; 7 Robinson Research Institute, Adelaide Medical School, University of Adelaide, Adelaide, SA Australia

**Keywords:** Health care, Medical research, Risk factors

## Abstract

Early newborn care provided in the first 2 days of life is critical in reducing neonatal morbidity and mortality. This care can be used to monitor and evaluate the content and quality of neonatal postnatal care. This study aimed to identify determinants and geographic distributions of early newborn care uptake in Ethiopia. We used data from the 2019 Ethiopian Mini Demographic and Health Survey (EMDHS). We conducted a multilevel binary logistic regression model and geographic analysis to identify the determinants of receiving early newborn care. A total of 2105 children were included in the study. Of the included children, 39.6% (95% confidence interval (CI) 38%, 42%) received at least two components of early newborn care services in the first 2 days after birth. Greater odds of receiving early newborn care were experienced by infants to mothers with secondary or above education (adjusted odds ratio (AOR) = 1.72; 95% CI 1.44, 2.18), from households with highest wealth quantiles (AOR = 1.47; 95% CI 1.16, 1.79), with at least one antenatal care contact (AOR = 2.73; 95% CI 1.79, 4.16), with birth at health facility (AOR = 25.63; 95% CI 17.02, 38.60), and those births through cesarean section (AOR = 2.64; 95% CI 1.48, 4.71). Substantial geographic variation was observed in the uptake of early newborn care in Ethiopia. Several individual- and community-level factors were associated with newborn postnatal care. Policymakers should prioritise these areas and the enhancement of postnatal healthcare provisions for mothers with low socioeconomic status.

## Introduction

The immediate postnatal period, particularly the first 48 h, is crucial for women, newborns, and families. This period has a range of physical, social, and emotional changes that require special attention and care^1^. The first 2 days of life are critical, as many neonatal deaths occur during this time^2,3^. Postnatal care for a newborn during this period can significantly reduce neonatal mortality^4^ as it allows for identifying early newborn complications and initiating appropriate care and treatment timely^2^.

The World Health Organization (WHO) recommends that all newborns should receive a health check within 2 days after birth to identify newborn complications and initiation of appropriate care and treatment^5^. However, globally the median coverage for routine newborn postnatal care within the first 2 days after birth is 64% with significant variations among countries^5^. For example, while the 2016 Ethiopian Demographic and Health Survey reported that only one-fourth (27%) of newborns received postnatal care within 2 days^6^, the corresponding rate was higher in Ghana (81%)^7^ and Rwanda (75%)^8^. Globally, three-fourths (74.3%) of the total neonatal deaths occur in the first week of life, and 40% of neonatal deaths occur within 24 h after birth^9,10^. In Ethiopia, more than half (52.4%) of neonatal deaths occurred within the first 2 days of birth^11^.

Postnatal newborn care efforts need to expand beyond coverage and survival of the neonate to include quality care^5^. The revised WHO guidelines for 2022 recommend the provision of quality postnatal care as one of the components of maternal and child health care^5^. Measuring temperature, counseling on danger signs, counseling and observation of breastfeeding, and weighting of the child are among the recommended early newborn care services^12^. However, postnatal care programs are among the weakest of all reproductive health programs in low and middle-income countries such as Ethiopia^2,13^. There is minimal evidence on the magnitude and determinants of newborn postnatal care uptake within 2 days after birth in Ethiopia^14^. Moreover, updated evidence on the uptake of essential components of newborn postnatal care and its distribution is needed. Therefore, this study aims to identify determinants of receiving signal components of early neonatal postnatal care and explore geographic distributions in Ethiopia. This will be important for policymakers to identify priority areas for interventions to increase newborn postnatal care.

## Methods

### Data source, study setting, and period

The DHS Kid’s Records (KR) datasets were extracted from 2019 EMDHS^15^. Ethiopia is an East African country located 3°–14° N and 33°–48°E with 1.1 million sq. km coverage. It is Africa's second most populous country and is federally decentralized into eleven regions and two city administrations currently^16,17^. Four regions (Sidama, Central Ethiopia, South Ethiopia and Southwest Ethiopia) have been formed from Southern Nations, Nationalities, and Peoples' Region (SNNPR) after the survey has been conducted^16,18^, and the results were presented as part of the SNNPR region.

### Participants and sample size

**Figure 1:**
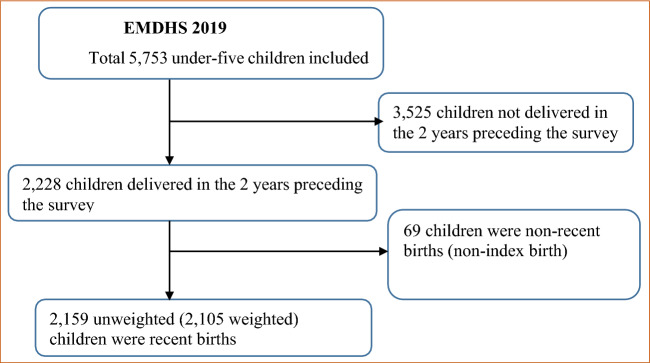
Final sample size and schematic presentation of the sample selection.

Participants were children who were born in the 2 years prior to the survey period. Of all mothers with children under 5 years in the 2019 EMDHS (n = 5753), those women with recent live births in the past 2 years from the date of interview (n = 2228) were asked for the provision of neonatal postnatal care services. Finally, a total of 2105 weighted samples were included for the analysis (Fig. 1).

### Sampling technique

A stratified two-stage cluster sampling method was used for data collection. From the total 11 regions in Ethiopia, ten administrative regions were stratified by dividing them into urban and rural areas except for Addis Ababa, and hence a total of 21 sampling strata were created. From these strata, a total of 305 clusters or Enumeration Areas (EAs) (93 in urban areas and 212 in rural areas) were selected (based on the census frame created for the 2019 Ethiopian Population and Housing Census (EPHC). To ensure that survey precision was comparable across regions, sample allocations were done through an equal allocation where 25 clusters were selected from each of six regions (Tigray, Afar, Somali, Benishangul Gumuz, Gambella, and Harari) and two city administrations (Addis Ababa and Dire Dawa) with the total of 200 clusters. However, 35 clusters were selected from each of the three larger regions: Amhara, Oromia, and the SNNPR with a total of 105 clusters. Clusters or EAs are geographic areas covering an average of 131 households^17,19^. To account for the differences in population sizes between regions, sample weighting was applied during the analysis.

### Outcome variable

Early newborn care was considered when a mother report recipient of at least two of the following five basic neonatal services within the first 2 days after birth^2,17^: (i) examining the cord, (ii) measuring the newborn's temperature, (iii) counseling on the newborn’s danger signs such as feeding problems, reduced activity, difficult breathing, fever, fits or convulsions, and cold skin, (iv) counseling on breastfeeding, and (v) observing breastfeeding.

### Independent variables

We considered individual and community level factors. The individual level risk factors included socio-demographic characteristics such as the age of the mother/caregiver, education status, marital status, occupation, family size, wealth index, and religion. Wealth status was created using principal components analysis and coded as lowest, low, middle, high, and highest in the EMDHS dataset^20^. Child-related determinants included birth order, sex, age, and the number of children in the family aged under-5 years. Health service utilization factors included the place of delivery, mode of delivery, and antenatal care (ANC) visits. At the community level, we included determinants such as place of residence, administrative region, and community-level women illiteracy. Based on the development status and the need for governmental support, the 11 regions of Ethiopia are categorized into three groups; 'three Metropolis' (Addis Ababa, Harari, and Dire Dewa), large central (Tigray, Amhara, Oromia, SNNPR), and “communities with predominately pastoralist regions” (Afar, Benishangul-Gumuz, Gambelia, and Somali)^21^. Moreover, community-women illiteracy rate was defined by the percentage of women that could not read or write^22^. Community illiteracy was categorised based on the 2017 national illiteracy cut-off (i.e., 55.6%) and it was regarded as high when the proportion of women in a cluster who were not able to read or write was greater than or equal to 55.6% and was regarded low otherwise^22,23^. These variables were considered based on previously published literature and the availability of information in the dataset (Supplementary Table 1).

### Statistical analysis

The generalised linear mixed model (GLMM) was employed for this study, in which the linear predictor comprises both random and fixed effect analyses. Multilevel binary logistic regression analyses were conducted. Variables with a *p*-value < 0.20 in the bivariable analysis were considered eligible for the multivariable analysis. Associations between dependent and independent variables were assessed, and their strength was presented using adjusted odds ratio (AOR) and 95% confidence intervals, with statistical significance set at a *p*-value < 0.05.

Four models were developed: the first model (null model) only included the cluster level using random intercepts. The second model (Model 2) included the individual-level variables plus random intercept, the third model (Model 3) included the community-level variables plus random intercept and the fourth model (Model 4) included both individual- and community-level variables plus random intercept.

The measure of variation or random effects was estimated by the median odds ratio (MOR), intra class correlation coefficient (ICC), and Proportional Change in Variance (PCV). The ICC, which reveals the variation in receipt of early newborn care between clusters, is calculated as $ICC=\frac{\sigma 2}{(\sigma 2+\pi /3)}*100\%$ ;$ICC=\frac{\sigma 2}{(\sigma 2+3.29)}*100\%$. Where $\sigma 2$ indicates cluster variance. Based on this, in our model, 46% of the variation in the receipt of early newborn care was due to regional variation, with the remaining 54% being within-cluster. The MOR is defined as the median value of the odds ratio of receipt of early newborn care between the area at the highest risk and the area at the lowest risk when randomly picking out two clusters (EAs). The MOR in this study was calculated as MOR = exp (√(2 × $\sigma 2$) *0.6745), or ${\text{MOR}}={\text{exp}}(0.95*\sigma )$, Where, $\sigma 2$ indicates that cluster variance^24–26^.

In our study, the MOR between the higher and lower areas when randomly picking out two clusters of early newborn care among clusters was 4.91 (95% CI: 3.87, 6.52) in the first model. The PCV reveals the variation in early newborn care among children under 2 years of age explained by factors. The PCV is calculated as; $PCV=\frac{\text{Variance of null model-Variance of full model} }{\text{Variance of null model}}*100\%$^24–26^. Moreover, in this study, about 53.38% of the variation in early newborn care in children was explained by the final model (model four).

Deviance was used for model comparison, and the model with the lowest deviance (Model 4) was considered the best-fit model. We also used the likelihood ratio test (LRT)—a statistical test used to compare the fit of two models (simpler model vs a more complex model). A significant p-value of LRT in all our models suggested that the more complex model (multi-level analysis) provided a significantly better fit to the data than the simpler model (standard logistic regression model). Moreover, based on the Variance Inflation Factors (VIF) results, there was no multicollinearity between independent variables in all models (Table 1). All analyses were conducted by applying sample weighting to account for probability sampling and non-response to restore representativeness.

**Table 1:** Parameters and model fit statistics for multilevel regression analysis models.

Parameters	Model 1 (null model)	Model 2	Model 3	Model 4
Cluster level variance (σ2)	2.81	1.37	2.13	1.31
ICC	0.46	0.30	0.39	0.28
MOR	4.91 (95% CI 3.87, 6.51)	3.04 (95% CI 1.67, 4.41)	4.01 (95% CI 2.87, 5.52)	2.96 (95%CI 1.65, 4.27)
PCV	Reference	51.3%	24.2%	53.4%
Model fitness				
Deviance	2322	1738	2256	1728
LRT	504 (*P*-value < 0.001)	151(*P*-value < 0.001)	149 (*P*-value < 0.001)	147 (*P*-value < 0.001)
Largest VIF		2.39	1.73	2.86

ICC, Inter cluster Correlation Coefficient; MOR, Median Odds Ratio; PCV, Proportional Change in Variance; VIF, Variance Inflation Factors; LRT, Likelihood ratio test.

### Spatial analysis

The Global Moran’s I statistic was used to assess spatial autocorrelation^27^. The Global Moran's I value ranges from − 1 to + 1, where a value below 0 indicates negative spatial autocorrelation and values above 0 indicate positive spatial autocorrelation. Moreover, having ≥ 0.8 indicates a very strong autocorrelation, 0.5 to 0.7 moderate, and < 0.5 indicates a weak one^27,28^. Whereas a spherical semivariogram ordinary kriging type spatial interpolation technique was used to predict the early newborn care among under 2 years children in Ethiopia for unsampled areas based on sampled clusters. The proportion of children who receive early newborn care in each cluster was taken as input for spatial prediction.

Using Kuldorff’s SaTScan version 9.6 software was used to fit Bernoulli-based modeled spatial scan statistics to identify the locations of clusters for lack of early newborn care^29^. The scanning window that moves across the study area in which children who received early newborn care were taken as cases and those children who did not receive them were taken as controls to fit the Bernoulli model.

### Ethical approval

Ethical approval was not required for this study as we used a secondary analysis of a publicly available survey from the Demographic and Health Survey (DHS) program.

## Results

### Mothers or caregivers and children's socio-demographic characteristics.

A total weighted sample of 2105 under-2 years children was included in this study. Nearly half (49%) of mothers of children were found between 25 and 34 years with a median age of 27 years (interquartile range (IQR) = 7 years). Nearly half (46%) of mothers had no formal education. There are nearly equal distributions of samples based on the wealth index categories (Table 2).

**Table 2:** Socio-demographic characteristics of the mothers/caregivers and the children in a study of early newborn care and determinants among under-two years old children in 2019 EMDHS (n = 2105).

Variables	Categories	Frequency	Percentage
Socio-demographic variables			
Age of women (years)	15–24	682	32.4
	25–34	1037	49.2
	36–49	386	18.4
Educational attainment of women	No education	978	46.4
	Primary education	840	40.0
	Secondary or above	287	13.6
Marital status of the mother	Currently married	1982	94.2
	Not currently married	123	5.8
Wealth index	Lowest	460	21.9
	Low	449	21.4
	Middle	393	18.7
	High	364	17.3
	Highest	439	20.8
Religion	Orthodox	753	35.8
	Muslim	758	36.0
	Protestant	555	26.4
	Others*	39	1.8
Child and health services-related variables			
Birth order	≤ 3rd	1221	58.0
	> 3rd	884	42.0
Sex of child	Male	1084	51.5
	Female	1021	48.5
Age of child (months)	0–5	662	31.4
	6–11	414	19.7
	12–23	1029	48.9
Number of under-five children in the family	One	893	42.4
	Two	945	44.9
	Three and above	267	12.7
Place of birth	Home	968	46.0
	Health institution	1137	54.0
Mode of birth	Others**	1973	93.8
	Cesarean section	132	6.2
ANC visits	No ANC visit	549	26.1
	At least one visit	1556	73.9
Community level variables			
Residence	Urban	553	26.3
	Rural	1552	73.7
Regions	Metropolis	83	3.9
	Large centrals	1824	86.7
	Pastoralist regions	198	9.4
Community-women illiteracy	Low	1859	88.3
	High	246	11.7

Other*—Religions such as catholic, traditional, and other religions. ANC—Antenatal care.

Others**—Mode of delivery such as spontaneous vaginal delivery, instrumental assisted delivery, and other delivery methods.

### Uptake of early newborn care among under 2 years children in Ethiopia

**Figure 2:**
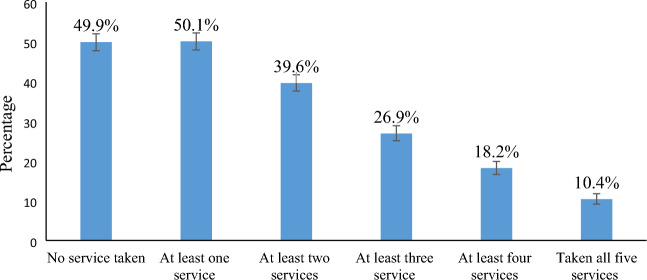
Proportion of early newborn care among under-two children in Ethiopia, using 2019 EMDHS.

The proportion of early newborn care among under-two children in Ethiopia was 39.6% (95% CI 38%, 42%) and ranged from 18% in Somali to 90% in Addis Ababa region. While half of under 2 years of children (50%) received at least one early newborn care service within the first 2 days, only one in ten (10%) of newborns received all the available early newborn care (Fig. 2).

**Figure 3:**
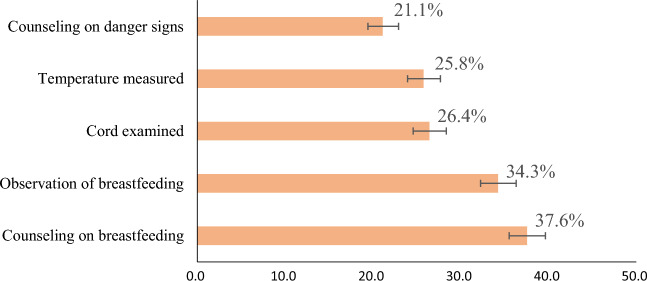
Proportions of early newborn care among under-two children in Ethiopia based on the types of care given, using the 2019 EMDHS.

Based on the recipient of each type of early newborn care, while 38% of mothers with under two children received counseling on breastfeeding, one-fifth (21%) of mothers with under 2 years children received counseling on newborn danger signs (Fig. 3).

### Determinants of early newborn care among under two age children in Ethiopia

Based on the chosen model (model 4), the education status of the mother/caregiver, wealth index of the household, age of the child, place of delivery, mode of birth, having ANC visit, residence, and region they live in, and community women illiteracy status were significant determinants (Table 3).

**Table 3:** Multilevel analysis of factors associated with early newborn care among children aged 0–23 months in Ethiopia: based on 2019 EMDHS (n = 2105).

Variables	Categories	Model 2^$^ AOR (95% CI)	Model 3 AOR (95% CI)	Model 4 AOR (95% CI)
Socio-demographic characteristics				
Age of women (years)	15–19	1.00		1.00
	20–35	0.99 (0.69, 1.40)		0.93 (0.65, 1.32)
	36–49	1.52 (0.90, 2.56)		1.42 (0.84, 2.40)
Educational attainment of women	No education	1.00		1.00
	Primary education	1.10 (0.78, 1.55)		1.08 (0.77, 1.52)
	Secondary or above	1.76 (1.47, 2.25) **		1.72 (1.44, 2.18) *
Marital status of the mother	Married	1.00		1.00
	Not married	2.31 (0.73, 2.35)		1.30 (0.73, 2.35)
Wealth index	Lowest	1.00		1.00
	Low	0.90 (0.55, 1.47)		0.84 (0.50, 1.39)
	Middle	0.78 (0.47, 1.31)		0.72 (0.43, 1.22)
	High	0.74 (0.43, 1.25)		0.65 (0.38, 1.12)
	Highest	1.40 (1.06, 1.75) *		1.47 (1.16, 1.79) *
Religion	Orthodox	1.00		1.00
	Muslim	1.04 (0.65, 1.67)		1.13 (0.67, 1.92)
	Protestant	0.37 (0.23, 0.61)		0.39 (0.24, 0.64)
	Others^β^	0.35 (0.69, 1.67)		0.35 (0.71, 1.76)
Child related characteristics and health service utilization of the mothers				
Birth order	≤ 3^rd^	1.00		1.00
	> 3^rd^	1.02 (0.69, 1.54)		1.07 (0.72, 1.61)
Sex of child	Male	1.00		1.00
	Female	1.14 (0.87, 1.49)		1.15 (0.78, 1.67)
Age of child	0–5	1.00		1.00
	6–11	1.14 (0.78, 1.68)		1.14 (0.77, 1.67)
	12–23	1.53 (1.12, 2.11) *		1.34 (0.91, 1.93)
Number of under 5 children	One	1.00		1.00
	Two	1.14 (0.84, 1.53)		1.11 (0.82, 1.51)
	Three and above	1.72 (1.01, 2.93)		1.68 (0.98, 2.96)
Place of delivery	Home	1.00		1.00
	Health institution	25.44 (16.92, 38.24) *		25.63 (17.02, 38.60)*
Mode of birth	Others^c^	1.00		1.00
	Cesarean section	2.81 (1.59, 4.98) *		2.64 (1.48, 4.71)*
ANC visits	No ANC visit	1.00		1.00
	At least one visit	2.75 (1.81, 4.18) *		2.73 (1.79, 4.16)*
Community level variables				
Residence	Urban		1.00	1.00
	Rural		0.36 (0.18, 0.73) **	**0.69 (0.47, 0.91)**
Region	Metropolis		1.00	1.00
	Large centrals		0.37 (0.14, 0.94) *	0.49 (0.19,1.28)
	Pastoralist regions		0.16 (0.06, 0.44) *	**0.32 (0.12, 0.93)**
Community-women illiteracy	Low		1.00	1.00
	High		0.47 (0.22, 0.72) *	0.51 (0.32, 0.80)**

*p-value < 0.05. **p-value < 0.01. AOR, Adjusted Odds Ratio. CI, Confidence Interval, ANC visit, Antenatal care visit.

Others ^β^: Religions such as catholic, traditional, and other religions.

Others ^c^: Mode of delivery such as spontaneous vaginal, instrumental delivery.

^**$**^Model 1(null model); the model which contains only the dependent variable.

Significant values are in bold.

Children born from women with secondary or above education have 1.72 times greater odds of receiving early newborn care than those born from women without formal education (AOR = 1.72; 95% CI 1.44, 2.18). On the other side, children from high-community women illiteracy clusters have 49% lower odds of early newborn care uptake than those from low-community women’s illiteracy status (AOR = 0.51; 95% CI 0.32, 0.80).

Moreover, children born from women who had at least one ANC visit (AOR = 2.73; 95% CI 1.79, 4.16) and who were born at a health facility (AOR = 25.63; 95% CI 17.02, 38.60) had 3- and 26-times higher odds of receiving early newborn care as compared to those not having ANC visits and home births respectively. In addition, the odds of receiving early newborn care among mothers who delivered through cesarean section were 2.64 times higher than those with other delivery modes (AOR = 2.64; 95% CI 1.48, 4.71).

Children from households with highest wealth quantiles have 47% higher odds of receiving early newborn care than children from households with highest wealth quantiles (AOR = 1.47; 95% CI 1.16, 1.79). The odds of receiving early newborn care among children who resided in rural areas and communities with predominately pastoralist regions are 31% and 68% lower than their counterparts (AOR = 0.69; 95% CI 0.47, 0.91) and (AOR = 0.32; 95% CI 0.12, 0.93) respectively (Table 3).

### Geographic analysis of early newborn care among under two aged children

**Figure 4:**
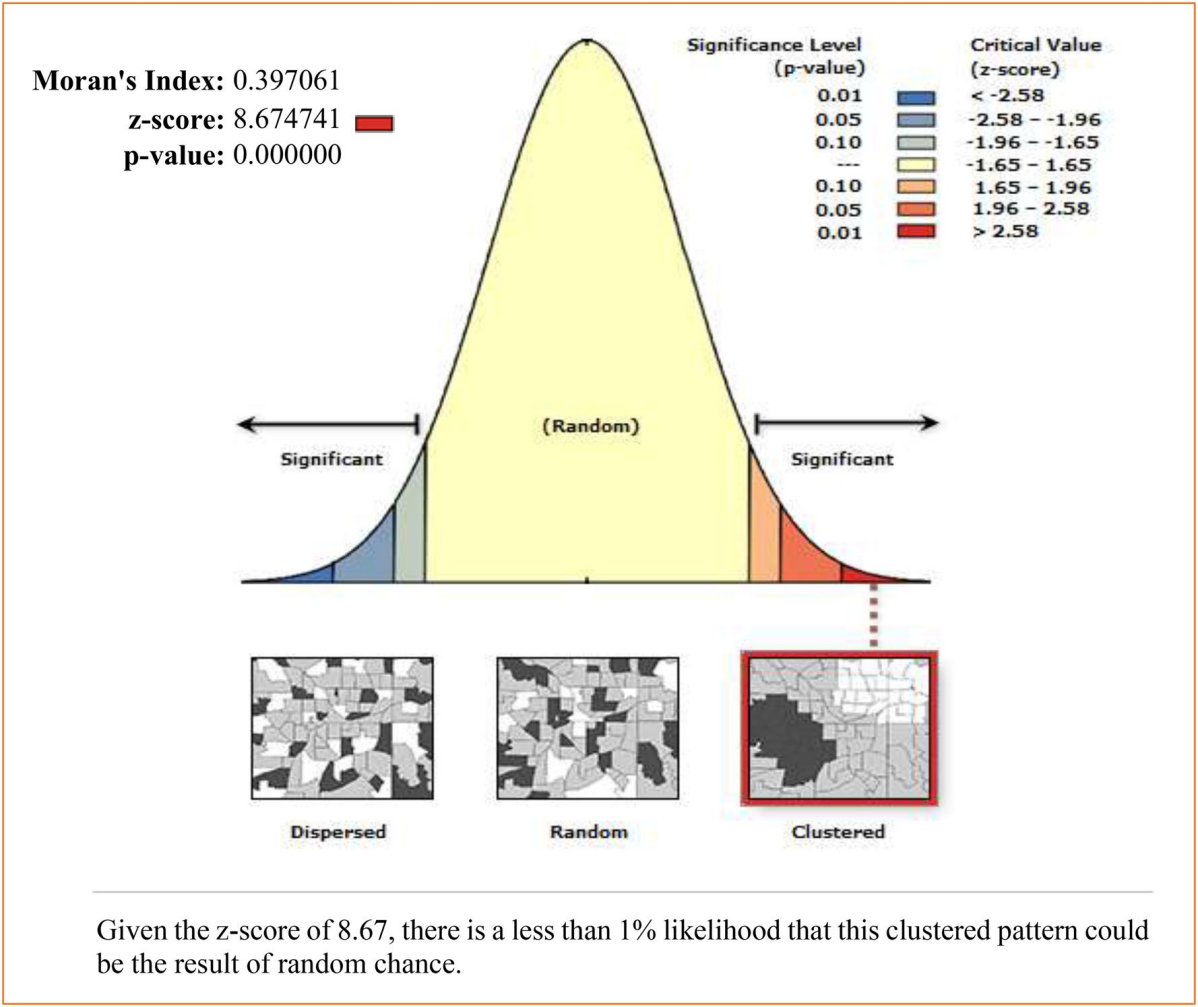
Spatial autocorrelation of early newborn care among under-two children in Ethiopia 2019 EMDHS. The base map for the shapefile was sourced from: https://gadm.org/download_country.html#google_vignette.

**Figure 5:**
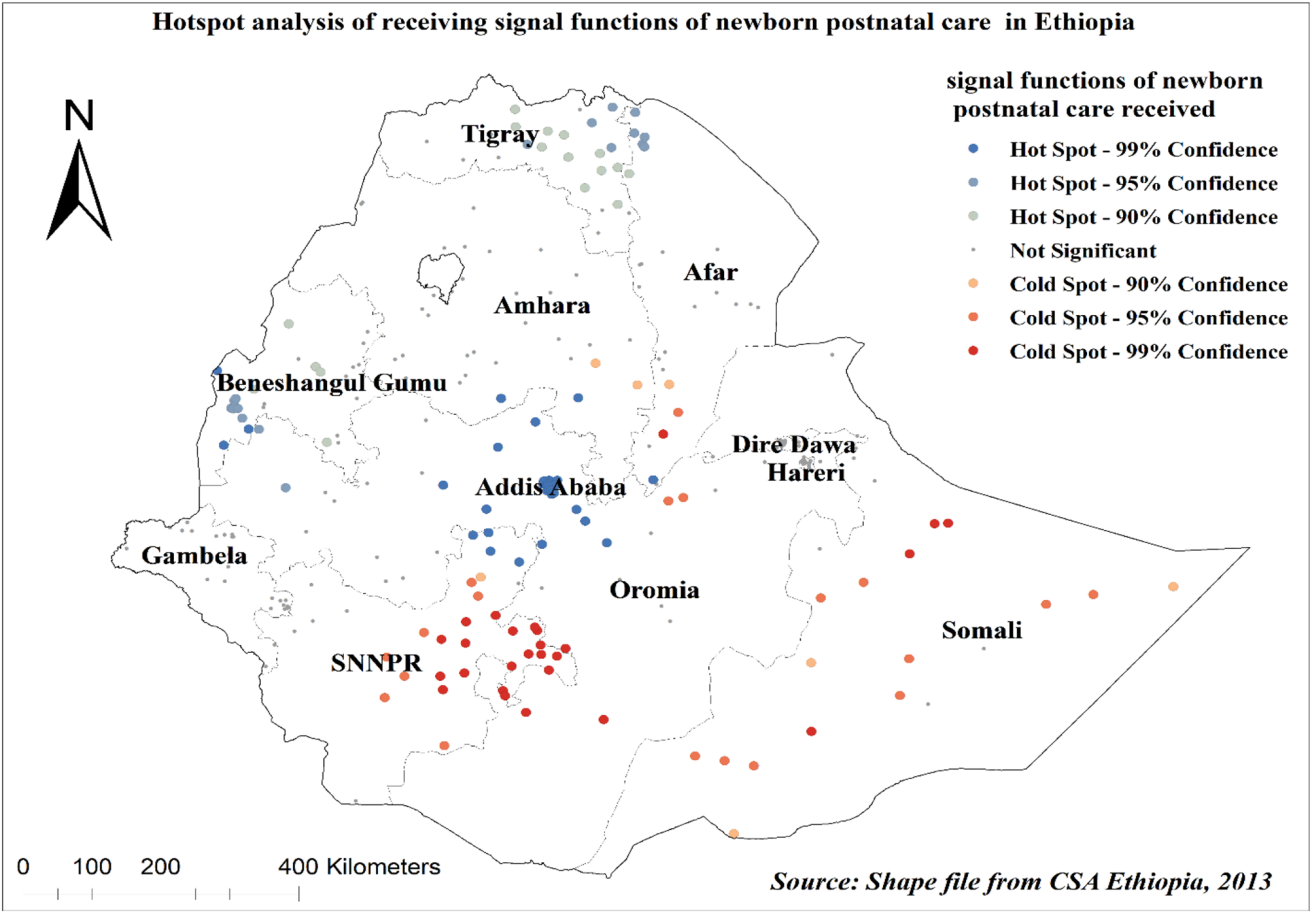
Hotspot analysis of early newborn care after birth among under-two children in Ethiopia, 2019 EMDHS. The base map for the shapefile was sourced from: https://gadm.org/download_country.html#google_vignette.

The spatial autocorrelation results of taking early newborn care in Ethiopia showed significant positive spatial autocorrelation over regions in the country. It was found to be clustered with Global Moran's Index value: 0.3971 with (*p* < 0.001) (Fig. 4). Early newborn care was more practiced in Addis Ababa, Tigray, and Benishangul Gumuz regions, whereas the SNNPR, Somali, and southwest Oromia regions are the cold spot areas (Fig. 5).

**Figure 6:**
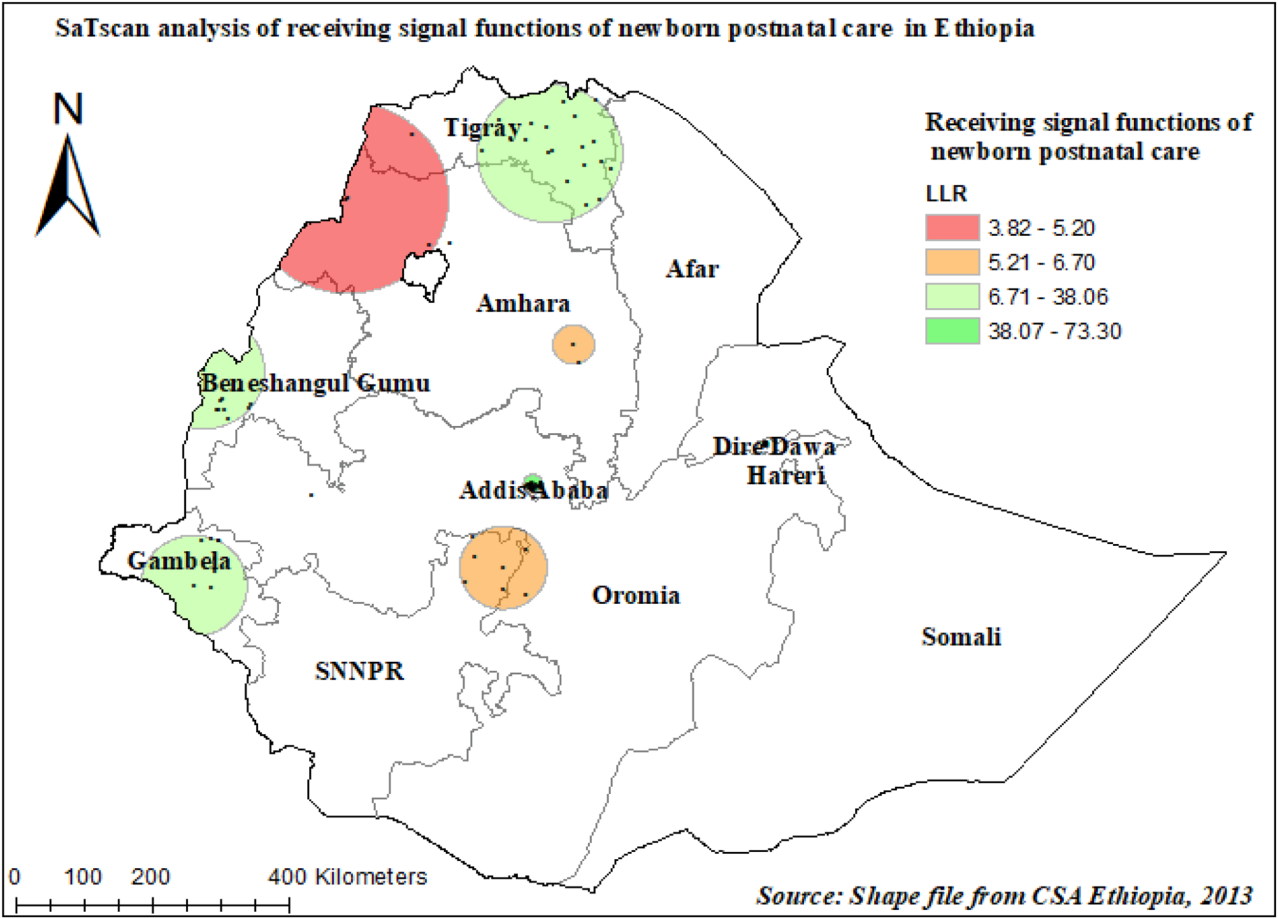
SaTscan analysis of early newborn care among under-two children in Ethiopia, 2019 EMDHS. The base map for the shapefile was sourced from: https://gadm.org/download_country.html#google_vignette.

The SaTscan analysis of early newborn care among children in Ethiopia showed that 21 primary clusters and 31 secondary and other clusters were detected for having early newborn care. The primary clusters were centered at 9.066209 N and 38.754639 with a 13.96 km radius and located in Addis Ababa. Children who were found in the primary window were 2.5 times more likely to get early newborn care than out in-window regions (RR = 2.45, *P*-value < 0.001) (Table 4, Fig. 6).

**Table 4:** Significant spatial clusters of early newborn care among under-two children in Ethiopia, 2019 EMDHS.

Clusters	Enumeration areas (clusters) detected	Coordinate/radius	Population	Cases	RR	LLR	*P*-value
1ry (21)	256, 265, 266, 257, 258, 263, 267, 264, 262, 261, 260, 273, 276, 259, 268, 275, 271, 270, 269, 272, 277	9.066209 N, 38.754639 E/13.96 km	98	95	2.45	73.29	< 0.001
2nd (8)	146, 157, 151, 149, 147, 152, 153, 154	10.589922 N, 34.352539 E/88.60 km	65	60	2.28	38.06	< 0.001
3rd (8)	206, 214, 213, 212, 230, 211, 209, 208, 2, 14, 11, 13, 17, 12, 23, 5, 7, 16, 3	7.628705 N, 34.263138 E/77.43 km	221	161	1.71	24.48	< 0.001
4th (15)	293, 295, 292, 294, 290, 291, 297, 288, 285, 286, 289, 287, 283, 296, 302	9.591016 N, 41.875191 E/4.24 km	82	54	1.59	22.73	< 0.001

**Figure 7:**
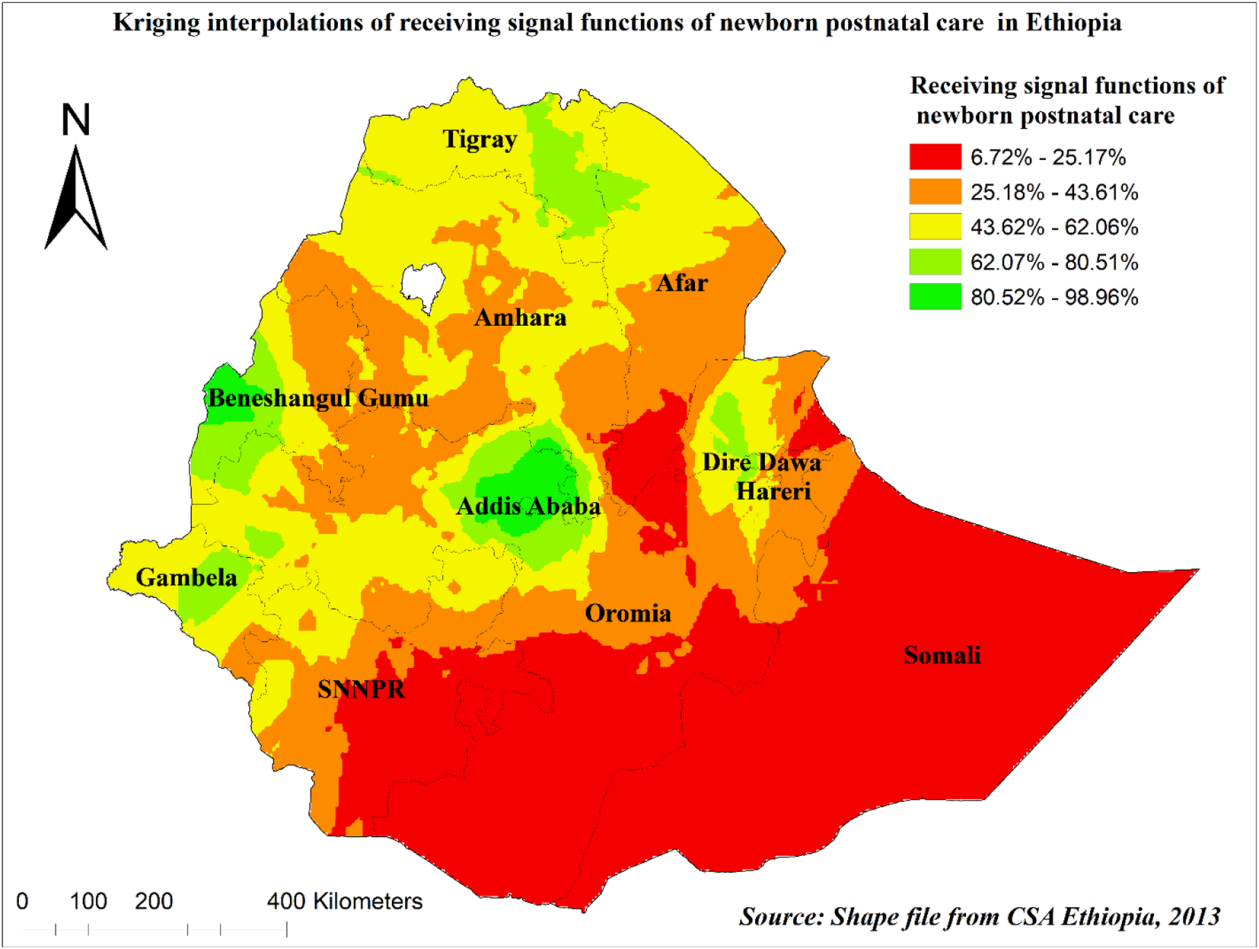
Kriging interpolation of early newborn care among under-two children in Ethiopia, 2019 EMDHS. The base map for the shapefile was sourced from: https://gadm.org/download_country.html#google_vignette.

The Kriging interpolation methods of predicting early newborn care among children in Ethiopia showed that high-risk areas predicted early newborn care ranging from 80.52% to 98.96% and are in Addis Ababa and Benishangul Gumuz regions. The lower predicted area was seen in Somali, Southern Afar, Southern SNNPR, and Oromia regions ranging from 6.72% to 25.17% (Fig. 7).

## Discussion

This study aimed to identify determinants of early newborn care and geographic variations in services uptake in Ethiopia. Despite the WHO recommends all newborns to receive early newborn care^30^, we found a substantially low uptake of early newborn care in Ethiopia, with only two out of five births (40%) receiving early neonatal care. Several individual and community-level factors were associated with low uptake of early neonatal care. There was a significant spatial variation in receiving care in the country.

This proportion of early newborn care in our study was lower than the report from Ghana (84.8%)^7^, Rwanda (85.4%)^8^, Uganda (50%)^31^, and Nepal (66%)^32^. The low proportion of early neonatal care might be due to the difference in healthcare access. For instance, Ethiopia's universal health effective coverage was 38% in 2019 compared with 45% in Ghana^33^. In addition, the awareness difference in the population towards the benefit of postnatal checkups^34^ could be another reason. Moreover, the sociocultural difference in healthcare utilization could have contributions^31,32^. For example, a study in Bangladesh showed that there was a strong restriction of movement of mother and baby outside the home during the early postnatal period^35^.

Even though substantially low uptake of early newborn care observed in this study, Ethiopia has made significant progress from 27% in 2016 to nearly 40% in 2019^36^. One major contributing factor to this progress could be the reduction in home deliveries across the country, which decreased from 73% in 2016 to 48% in 2019^2^. This improvement in facility-based deliveries has likely led to increased access to postnatal care services for new mothers, contributing to better health outcomes for both mother and child^2^. Moreover, in recent years efforts have been made to increase maternal health service utilization^37,38^.

In this study, while half (50%) of the under 2 years of children visited postnatal care services and received at least one component of early newborn care, the proportion of newborns who received all component of early newborn care was only 10%, reflecting the low implementation of newborn postnatal care services and representing a missed opportunity that newborns were not receiving the recommended services despite they were brought to health facilities. This is supported by a study conducted in the northern Ethiopia, which showed the quality of postnatal care offered to clients in the hospitals of Tigray was poor and below standard^39^. This was due to lack of essential equipment, postnatal care training, and workload^39^. The midwives did not take any training on postnatal care and resources were inadequate for provision of comprehensive and quality postnatal care^39^. The gap in the provision of limited essential services was also observed in another study in Ethiopia when only 22% of women visiting ANC provided essential components of ANC of 75% of these women attended ANC clinics. Strengthening the quality of care as well as ensuring the availability of quality health services for newborns are required for accelerating progress on neonatal survival and infant health and well-being^40^.

In our study, newborns whose mothers attended secondary or above education had higher odds of early newborn care than women without formal education. Similarly, at the community level, children from communities with higher women illiteracy rate had fewer odds of early newborn care than those from low illiteracy level communities. This is consistent with a study from Nepal^32^. This is because educated women are more likely to have visited health facilities with a better quality health services^41,42^.

In our study, mothers who had at least one ANC visit during the pregnancy period have higher odds of early newborn care as compared to those not having an ANC visit. This is supported by other studies in Ethiopia^43,44^ and Pakistan^45^. Mothers who attend ANC may be more aware of the importance of postnatal care for their newborns. During ANC visits, mothers receive counseling on the benefits of postnatal care, including education on the components of early newborn care that indicate a need for immediate medical attention. This information may contribute to improved knowledge among mothers regarding postnatal care^43,46^. Moreover, during ANC visits, comprehensive maternal and child care counseling has been given, and the importance of institutional delivery and postnatal checkup is provided^44,47^.

Moreover, children delivered at health institutions have higher odds of early newborn care than home-delivered mothers. This is supported by a study in Uganda^31^, and Ethiopia^14^. Moreover, in this study mothers who delivered through cesarean section were more likely to have immediate newborn postnatal care than other delivery modes. This is also in line with another study in Ethiopia^44^. This could be because mothers who gave birth at home are culturally restricted from moving out of their homes for a certain period, which reduces postnatal care utilisation^14^. Therefore, if the care is not given home to home, they might miss their visit^35^. Whereas mothers who gave birth in the hospital get advice and information about the postnatal care (PNC) service and eventually they might uptake the service^48^. Moreover, mothers who gave birth through cesarean section might stay in the health facility for 2 days. More postpartum care are also needed for these mothers than those who had vaginal birth^44^. Therefore, they could have a high chance of getting the service^14^.

This study revealed that children from households with highest wealth quantiles have higher odds of receiving early newborn care than the lowest wealth quantile family. This is supported by a systematic review and meta-analysis on inequities in postnatal care in low- and middle-income countries that reported significant variation in the use of postnatal care by socioeconomic status and geographical determinants^3,5^. This might result from the freedom to make autonomous decisions on the use of household incomes, and they can also afford payments related to health care services to their newborns and themselves^44,49^.

In addition to the individual factors discussed above, community-level factors are associated with early newborn care. The odds of receiving early newborn care among children who live in rural residences and communities in predominately pastoralist regions of Ethiopia were lower than in urban and metropolitan regions, respectively. The findings align with earlier Ethiopian studies^50–52^. It could also be the reason for high neonatal mortality in rural areas than in urban areas^11^.

Moreover, the spatial distribution of early newborn postnatal care was not random in our study. It was more practiced in Addis Ababa, Tigray, and Benishangul Gumuz regions, whereas the SNNPR, Somali, and southwest Oromia regions are the lower covered areas. This is supported by similar studies^14,53,54^. Spatial clustering of early postnatal non-utilization was observed in the Eastern, Southern, and southeastern parts of the country^14^. The study showed that mothers in the Tigray region have better access to health facilities and receive PNC. In addition, the Health Extension Workers (HEW) are motivated to visit the mothers at home disproportionally from the other regions^44^.

### Limitations

Despite newborn body weight is one of the essential components in the early newborn postnatal period, it was not collected in 2019 EMDHS, and hence we were not able to account in our analysis. Since the study subjects are under 2 years of children and asked their mothers about the recipient of early newborn care within the first 2 days, there might be a recall bias for relatively older children. The study also might have survivor bias since it includes living children at time of data collection from 2 days up to 2 years.

## Conclusions

The proportion of early newborn care in Ethiopia was low compared to WHO recommendation. Individual-level factors such as maternal education, wealth status of the household, and obstetric history was associated with postnatal care. Likewise, community-level variables such as residence, region, and community women illiteracy were associated with early newborn care. Moreover, the spatial distribution of newborn postnatal care was not random and better practiced in Addis Ababa, Tigray, and Benishangul Gumuz regions.

Health interventions should focus on improving the provision of quality and comprehensive maternal healthcare services to improve postnatal care, including through improving healthcare providers’ adherence to the provision of recommended early newborn care. Regions such as SNNPR, Somali, and southwest Oromia regions should be considered as priority areas for interventions to increase newborn postnatal care.

### Supplementary Information

Supplementary Table 1.

## Data Availability

Data from open databases are accessible to the general population. The website listed below allows access to it. https://dhsprogram.com/methodology/survey/survey-display-551.cfm.

## Abbreviations

AOR: Adjusted odds ratio
ANC: Antenatal care
CI: Confidence interval
CSA: Central statistical agency
DHS: Demographic and Health Survey
EMDHS: Ethiopian Mini Demographic and Health survey
GLMM: Generalized linear mixed model
ICC: Class Correlation Coefficient
KR: Kids record
LRT: Likelihood ratio test
MOR: Median odds ratio
PCV: Proportional change in variance
PNC: Postnatal care
SNNPR: Southern Nations, Nationalities, and Peoples' Region
VIF: Variance inflation factors
WHO: World Health Organization
