# Supplementary material for: Determinants and geographic distribution of early newborn care in Ethiopia: evidence from the 2019 Ethiopian Mini Demographic Health Survey
Source: Sci Rep. 2023 Dec 20;13:22690. doi: 10.1038/s41598-023-49812-9 (PMC10730702; doi:10.1038/s41598-023-49812-9)
Supplement: Supplementary file 1 — Supplementary Table 1. [file 41598_2023_49812_MOESM1_ESM.pdf]

Supplementary file

Table 1. Individual and community level independent variables in the study of determinant and geographic distribution early newborn care in Ethiopia.

Level	Variables	Measurements
Individual level variables	Age	The age of the mother/caregiver is categorized as 15-24, 25-34, and 36-49.
	Education level	Educational attainment is categorized as uneducated, primary, secondary, and above educational status.
	Marital status	The marital status of the mothers is categorized as married or not married.
	Occupation of women	The occupation of women is categorized as working and not working.
	Wealth index	It was created using principal components analysis and coded as lowest, low middle, high, and highest in the DHS data set.
	Religion	It was categorized as Orthodox, Muslim, Protestant, and Others (Catholic, traditional, and others).
	Birth order	Categorized as birth order less than or equal to three and above three.
	Sex of the child	The sex of the child is categorized as male or female.
	Age of the child	The age of the child is categorized as 0-5, 6-11, and 12-23 months.
	Number of under-five children in the family	Categorized as single, two and three, and more.
	Mode of delivery	Categorized as Cesarean Section and others
	Place of delivery	Classified as home delivery and health institution delivery
	ANC visit	Grouped as At least one ANC visit or not have ANC visit
Community level variables	Residence	Urban or rural based on where the household lives.
	Region	Based on the development status and the need for governmental support, the 11 regions of Ethiopia are categorized into three groups; 'three Metropolis' (Addis Ababa, Harari, and Diredewa), large central (Tigray, Amhara, Oromia, SNNPR), and " communities with predominately pastoralist regions" (Afar, Benshangul-Gumuz, Gambelia, and Somali) [20].
	Community-women illiteracy level	The illiteracy rate is defined by the percentage of the population of a given age group that can't read and write [21]. Community illiteracy level was categorized based on the 2017 national illiteracy level high if the proportion of illiterate women per cluster was 55.6%–100% and low if it was less than 55.6% [21 22].
